# An Evaluation of Soybean Protein Concentrate as a Replacement for Fish Meal with Methionine Supplementation in Diets for Hybrid Sturgeon (*Acipenser baerii* ♀ × *A. schrenckii* ♂)

**DOI:** 10.3390/ani15060787

**Published:** 2025-03-10

**Authors:** Zhaolin Li, Kai Xie, Jiufeng Gu, Xinyu Li, Yong Shi, Junzhi Zhang, Yi Hu, Xuezhi Zhu

**Affiliations:** 1 Fisheries College, Hunan Agricultural University, Changsha 410128, China; lizhaolin910829@163.com (Z.L.); xk971006@stu.hunau.edu.cn (K.X.); gujf4@haid.com.cn (J.G.); shiyong@stu.hunau.edu.cn (Y.S.); zhiun123@hunau.edu.cn (J.Z.); 2 Hunan Yuehai Feeds Group Co., Ltd., Changde 415000, China; lixinyu664476@163.com; 3 Key Laboratory of Breeding Biotechnology and Sustainable Aquaculture, Institute of Hydrobiology, Chinese Academy of Sciences, Wuhan 430072, China

**Keywords:** hybrid sturgeon, methionine, growth, liver health, intestinal health, muscle quality

## Abstract

Soy protein concentrate (SPC) is a cost-effective alternative to fish meal (FM) in aquaculture, but its deficiency in essential amino acids, particularly methionine, limits its application. This study evaluated the effects of methionine supplementation on growth, liver and intestinal health, and muscle quality in hybrid sturgeon (*Acipenser baerii* ♀ × *A. schrenckii* ♂) fed SPC-based diets. Four diets were formulated: an FM control diet, and SPC diets supplemented with 0% (M0), 0.25% (M2.5), and 0.50% (M5) methionine. Replacing FM with SPC without methionine (M0) significantly reduced weight gain and the protein efficiency ratio (PER) while increasing the feed conversion ratio (FCR) and hepatic lipid accumulation. Methionine supplementation (M5) restored growth performance, the PER, and muscle texture to levels comparable to the FM group. Intestinal enzyme activities (lipase and trypsin), villus height, and goblet cell counts significantly improved in the M5 group. Gene expression analysis showed that M5 upregulated tight junction genes (*claudin1*, *occludin*) and anti-inflammatory genes (*tgfβ*, *lysozyme*) while reducing pro-inflammatory cytokines (*il1β*, *il8*). In the liver, M5 reduced oxidative stress markers such as malondialdehyde (MDA) and improved antioxidant enzyme activities (SOD, CAT) while optimizing lipid metabolism, as evidenced by lower triglyceride (TG) and total cholesterol (TC) levels. Muscle quality analysis showed that M5 significantly increased muscle hardness, chewiness, and fiber density compared to M0. In conclusion, methionine supplementation at 0.50% effectively mitigates the negative effects of SPC, improving growth, liver and intestinal health, and muscle quality in hybrid sturgeon.

## Simple Summary

In this study, we formulated four different diets: an FM control diet, and SPC-based diets supplemented with 0%, 0.25%, and 0.50% methionine. Our findings demonstrate that replacing FM with SPC without methionine significantly impairs growth performance and protein efficiency, while increasing the feed conversion ratio and hepatic lipid accumulation. However, supplementation with methionine at 0.50% restored growth performance, the protein efficiency ratio, and muscle texture to levels comparable to the FM group. Furthermore, intestinal enzyme activities, villus height, and goblet cell counts were significantly enhanced with methionine supplementation, alongside improvements in liver oxidative stress markers, antioxidant enzyme activities, and lipid metabolism. Additionally, muscle quality parameters, including muscle hardness, chewiness, and fiber density, were significantly improved. Our findings offer insights into how methionine supplementation can effectively mitigate the negative effects of SPC in hybrid sturgeon, enhancing growth, liver and intestinal health, and muscle quality. These results have important implications for the sustainable use of SPC as a cost-effective alternative to FM in aquaculture.

## 1. Introduction

To address the issues of anti-nutritional factors in soybean meal replacing fish meal (FM), which negatively impact the growth, health, and quality of aquatic animals, researchers have increasingly turned to soybean protein concentrate (SPC) in recent years [1,2]. SPC, an upgraded alternative to soybean meal, has had most of its anti-nutritional factors removed [3]. However, despite these improvements, the issue of amino acid imbalance persists, particularly deficiencies in essential amino acids such as lysine and methionine, which continue to have adverse effects on aquatic animals [4]. In studies on rice field eel (*Monopterus albus*), growth performance was significantly reduced as FM was replaced with increasing levels of SPC in the diet, but this decline was alleviated by amino acid supplementation, highlighting the persistent imbalance in SPC’s amino acid profile [5,6]. Similarly, high levels of SPC replacement in diets have been shown to impair muscle quality, liver health, and intestinal integrity in species such as abalone (*Haliotis discus hannai*), golden crucian carp (*Cyprinus carpio* × *Carassius auratus*), swimming crab (*Portunus trituberculatus*), and white shrimp (*Litopenaeus vannamei*) [7,8,9,10].

Methionine is a sulfur-containing, non-polar, essential amino acid. Since fish cannot synthesize methionine internally, it must be obtained from external sources [11]. Methionine is a critical precursor for protein synthesis, and its adequate supply directly affects the growth rate and feed efficiency of fish. Studies on grass carp fry (*Ctenopharyngodon idella*), Yellow River carp (*Cyprinus carpio haematopterus*), and largemouth bass (*Micropterus salmoides*) have demonstrated that supplementing methionine significantly enhances the growth performance of fish [12,13,14]. Additionally, methionine participates in methylation processes and glutathione synthesis, which are crucial for liver and intestinal health. Research on largemouth bass and rice field eel has shown that methionine improves intestinal villus length and integrity, enhances nutrient absorption efficiency, and maintains intestinal barrier function [15,16]. Studies on white shrimp and Atlantic Salmon (*Salmo salar*) have found that appropriate methionine supplementation in the diet significantly improves a fish’s antioxidant capacity [11,17]. Furthermore, research on yellow croaker (*Larimichthys crocea*) has shown that supplementing methionine in soy protein concentrate-based diets significantly reduces the fat content in the liver and lowers the risk of fatty liver disease [18]. Supplementing methionine can increase the total protein content in fish muscle, making muscle fibers denser, thereby enhancing muscle hardness and elasticity. In cobia (*Rachycentron canadum*), methionine supplementation significantly improved protein synthesis rates in muscle, leading to firmer muscle texture [19]. Moreover, studies have shown that methionine supplementation improves muscle water retention in gibel carp (*Carassius auratus gibelio*) and rainbow trout (*Oncorhynchus mykiss*), making their flesh more tender [20,21].

Siberian hybrid sturgeon (*Acipenser baerii* ♀ × *A. schrenckii* ♂) is a high-quality hybrid breed obtained by crossing Siberian sturgeon (*A. baerii*) and Amur sturgeon (*A. schrenckii*). It grows rapidly, has high nutritional value in both meat and eggs, and offers good economic benefits. It is one of the main sturgeon species farmed in China [22]. Some studies have shown that the protein requirements of Siberian hybrid sturgeon are influenced by factors such as diet composition and growth stage, typically being around 40% [23,24]. The main animal protein source in sturgeon feed is FM; however, due to the limitations of FM use, increasing research has focused on replacing fish meal with soy protein in sturgeon diets. In a study [25] on shovel-nose sturgeon (initial weight: 175 g), feeding a diet with 51% soybean meal significantly reduced the specific growth rate and feed efficiency. The authors of [26] found that replacing more than 58% of fish meal with soy protein isolate in Amur sturgeon (initial weight: 26 g) negatively affected growth. A key factor in the use of soy protein concentrate (SPC) is a deficiency in essential amino acids, particularly methionine. Therefore, the main objective of this study is to evaluate the effects of supplementing methionine in SPC-based diets, replacing fish meal, on the growth performance, liver and intestinal health, and muscle quality of hybrid sturgeon, and to explore the role and optimization potential of methionine in SPC diets.

## 2. Materials and Methods

### 2.1. Experimental Diets

The experimental diets utilized fish meal (FM), soy protein concentrate (SPC), chicken meal, fermented soybean meal, soybean meal, and rapeseed meal as the main protein sources, with fish oil and soybean oil as lipid sources. The feed ingredients were sourced from Hunan Yuehai Feeds Group Co., Ltd. (Changde, China). Four different diets were formulated: the FM diet and three SPC replacement diets supplemented with 0%, 0.25%, and 0.50% methionine (designated as M0, M2.5, and M5, respectively). Before feed preparation, all ingredients were finely ground into powder and mixed stepwise in proportion to ensure uniformity. The premixed ingredients were blended in a V-shaped mixer for 5 min. Subsequently, 6% soybean oil was added to the mixtures, followed by water, to produce a uniform dough. The mixtures were pelletized using an extruder to produce sinking pellets with a diameter of 1.5 mm. Additional fish oil and 2% soybean oil were applied through spray coating. The diets were air-dried and stored in a cool environment until use. The detailed formulations and amino acid compositions of the diets are presented in Table 1, Table 2 and Table 3.

### 2.2. Feeding Experiment and Sample Collection

The study was conducted in the aquaculture system of an experimental base in Guangdong Province, China. Hybrid sturgeon (*Acipenser baerii* ♀ × *A. schrenckii* ♂) with an initial body weight of approximately 8 g were randomly allocated to experimental tanks after 24 h fasting to ensure uniformity and health. Thirty fish were stocked per tank, and four dietary treatments were tested with three replicates each, resulting in 12 cylindrical tanks (diameter 1.0 m; depth 0.5 m). Fish were fed twice daily at 6:30 and 17:00 with 2–5% of their body weight, with feed amounts adjusted every three days to ensure consumption within 10 min. The feeding trial lasted for eight weeks. Environmental parameters were strictly controlled, with water temperature maintained at 21.0 ± 0.5 °C, dissolved oxygen ≥ 6.0 mg/L, and ammonia nitrogen < 0.3 mg/L.

At the conclusion of the trial, fish were fasted for 24 h before sample collection. The fish in each tank were counted and weighed to calculate growth parameters. Nine fish were randomly selected from each tank for further analyses. Of these, three fish were anesthetized using eugenol and sampled for blood. Blood was drawn from the caudal vein using 1 mL syringes, placed in sterile tubes, allowed to clot overnight at 4 °C, and centrifuged at 3000 rpm for 10 min to obtain serum, which was stored at −80 °C. Another three fish were measured for body length and weight before being dissected on ice for liver and intestinal tissue collection. Liver and visceral weights were recorded. Intestinal tissues were washed with deionized water to remove contents and excess fat. A 1.5 cm segment of the mid-intestine was fixed in 4% paraformaldehyde for histological analysis. The remaining intestinal tissues were stored in 1.5 mL tubes at −80 °C. Muscle tissue (~1 cm^3^) was collected from the dorsal region, with one sample fixed in 4% paraformaldehyde and the other stored for texture analysis. The final three fish were preserved whole at −20 °C for body composition and amino acid analysis.

### 2.3. Analytical Methods

#### 2.3.1. Proximate Composition of Feed and Fish

The proximate composition of feed and fish samples was analyzed according to standard methods. Moisture was determined by drying samples to a constant weight at 105 °C (GB/T 6435-2014 [27]). Crude protein was measured using the Kjeldahl method with a fully automated Kjeldahl nitrogen analyzer (GB/T 6432-2018 [28]). Crude fat was determined using Soxhlet extraction with ether (GB/T 6433-2006 [29]). Ash content was analyzed by incinerating samples at 550 °C in a muffle furnace until constant weight was achieved (GB/T 6438-2007 [30]).

#### 2.3.2. Amino Acid Composition of Feed and Whole Fish

Amino acid composition was analyzed using high-performance liquid chromatography (HPLC) after the hydrolysis, filtration, evaporation, and derivatization of feed and dried whole fish samples (GB/T 18246-2019 [31]).

#### 2.3.3. Digestive Enzyme Activities

Intestinal samples were homogenized in different buffers: saline solution for amylase (AMS) and lipase (LPS) and a specific buffer for trypsin (TPS). The homogenates were centrifuged at 1160× *g* for 10 min, and the supernatants were stored for enzyme activity assays. Digestive enzyme activities were measured using commercial kits (Nanjing Jiancheng Bioengineering Institute, Nanjing, China) and a UV-5200 spectrophotometer.

#### 2.3.4. Histological Analysis of Intestinal and Muscle Tissues

Fixed intestinal and muscle samples were dehydrated, cleared, embedded in paraffin, sectioned, deparaffinized, and stained with hematoxylin–eosin (HE). Morphological structures were observed under an optical microscope equipped with a camera and analyzed using CellSen software (Ver.3.1.1). Measurements included intestinal villus height, muscular thickness, goblet cell counts, and muscle fiber density, which were quantified using ImageJ software (Ver.1.54f).

#### 2.3.5. Intestinal Gene Expression

RNA was extracted from intestinal tissues using Trizol. Approximately 30–50 mg of tissue was homogenized with 1 mL Trizol and zirconium beads, followed by chloroform extraction, isopropanol precipitation, and ethanol washing. The RNA pellet was dissolved in RNase-free water. RNA quality was assessed before reverse transcription using MonScriptTM RTIII Super Mix (Yeasen Biotechnology, Shanghai, China). Quantitative PCR was performed using SYBR^®^ Green qPCR Mix (Monad Biotech Co., Suzhou, China). The details of the methods were based on previous studies conducted in our laboratory [32]. Primer sequences are shown in Table 4.

#### 2.3.6. Serum Biochemical Analysis

Serum levels of triglycerides (TGs), total cholesterol (TC), high-density lipoprotein cholesterol (HDL-C), low-density lipoprotein cholesterol (LDL-C), glutamic oxaloacetic transaminase (GOT), and glutamate pyruvic transaminase (GPT) were determined using commercial kits (Nanjing Jiancheng Bioengineering Institute, China) and a Thermo-1510 microplate reader (Monad Biotech Co., Ltd., Suzhou, China).

#### 2.3.7. Hepatic Antioxidant Indicators

Liver tissues were homogenized in saline solution, and the homogenates were centrifuged at 1160× *g* for 10 min. Supernatants were analyzed for malondialdehyde (MDA), catalase (CAT), and superoxide dismutase (SOD) using commercial kits and a spectrophotometer.

#### 2.3.8. Muscle Texture Analysis

Muscle texture parameters, including hardness, adhesiveness, cohesiveness, elasticity, gumminess, and chewiness, were measured using TPA software (TMS-PRO, FTC, Sterling, VA, USA, Ver.6.1.26.0). Testing conditions included a compression speed of 30 mm/min with deformation set at 60% of the original length and an initial force of 0.1 N.

#### 2.3.9. Statistical Analysis

Data were processed using Excel 2019 and analyzed with R software (Ver.4.3.3). Normality (Shapiro–Wilk test) and homogeneity of variance (Bartlett’s test) were assessed. One-way ANOVA followed by Tukey’s HSD multiple comparisons or Student’s *t*-test was applied for normally distributed data (*p* < 0.05). Non-normal data were analyzed using the Kruskal–Wallis test or Wilcoxon rank-sum test, with Bonferroni correction for multiple comparisons. Orthogonal polynomial contrasts (OPCs) were performed to assess the linear and quadratic effects of methionine levels on FM-replacing SPC diets.

## 3. Results

### 3.1. Growth Performance

As shown in Table 5, compared with the FM group, the final body weight and weight gain rate of hybrid sturgeon in the M0 and M2.5 groups significantly decreased (*p* < 0.05), while the feed conversion ratio significantly increased, and protein efficiency significantly declined in the M0 group (*p* < 0.05). Compared with the M0 group, the final body weight, weight gain rate, and protein efficiency significantly improved, while the feed conversion ratio significantly decreased in the M5 group (*p* < 0.05). No significant differences in survival rates were observed among the groups (*p* > 0.05).

### 3.2. Physical Indices

As shown in Table 6, compared with the FM group, the hepatosomatic index of hybrid sturgeon significantly increased in the M0 and M2.5 groups, and the VSI significantly increased in the M0 group (*p* < 0.05). Compared with the M0 group, the VSI significantly decreased in the M2.5 and M5 groups. There were no significant differences in condition factors among the groups (*p* > 0.05).

### 3.3. Whole-Body Proximate Composition

As shown in Table 7, compared with the FM group, the crude protein content in the whole body of hybrid sturgeon significantly decreased in the M0 group (*p* < 0.05), while it significantly increased in the M5 group compared to the M0 group (*p* < 0.05). No significant differences were observed in whole-body crude lipid and ash content among the groups (*p* > 0.05).

### 3.4. Whole-Body Amino Acid Composition

As shown in Table 8, no significant differences were observed in amino acid composition among the groups (*p* > 0.05).

### 3.5. Intestinal Digestive Enzyme Activities

As shown in Table 9, compared with the FM group, the intestinal amylase activities of hybrid sturgeon significantly increased in the M0, M2.5, and M5 groups (*p* < 0.05). The intestinal lipase and trypsin activities of the M0 group significantly decreased compared to the FM group (*p* < 0.05), while these activities significantly increased in the M5 group compared to the M0 group (*p* < 0.05).

### 3.6. Intestinal Morphological Structure

**Figure 1:**
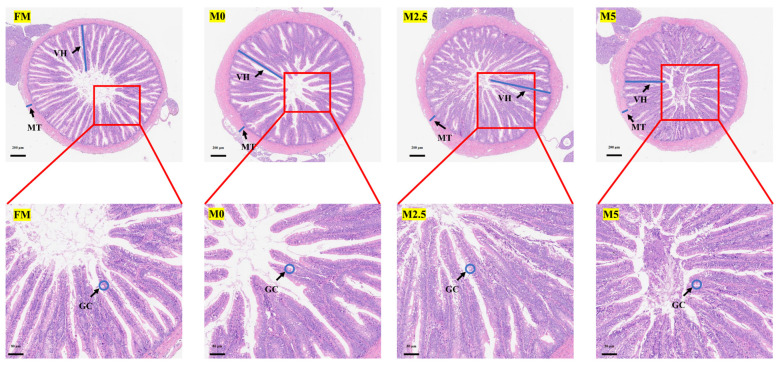
Effects of replacing fish meal with soybean protein concentrate and supplementing methionine on intestinal tissue structure of hybrid sturgeon (*A. baerii* ♀ × *A. schrenckii* ♂). Notes: H&E staining (×40; ×100). VH, MT, and GC represent intestinal villus height, muscle thickness, and goblet cells, respectively.

As shown in Figure 1 and Table 10, compared with the FM group, the villus height and goblet cell number in the intestine of hybrid sturgeon significantly decreased in the M0 group (*p* < 0.05). Compared with the M0 group, these parameters significantly increased in the M2.5 and M5 groups (*p* < 0.05).

### 3.7. Intestinal Barrier-Related Gene Expression

**Figure 2:**
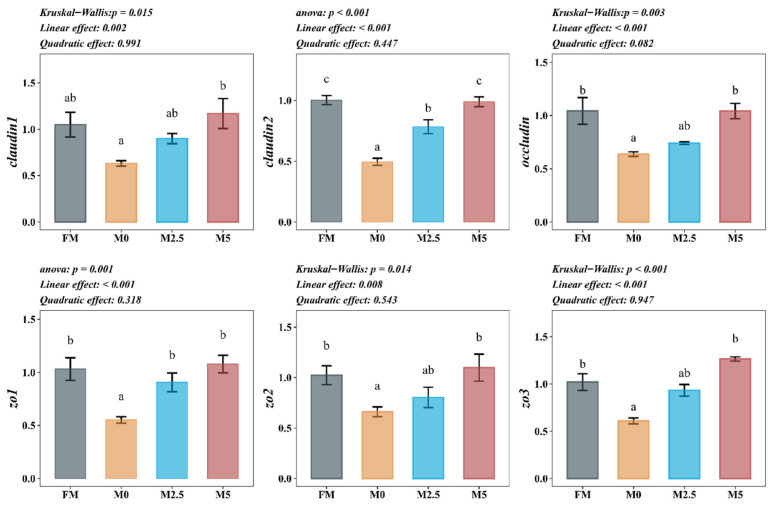
Effects of replacing fish meal with soybean protein concentrate and supplementing methionine on intestinal physical barrier-related gene expression of hybrid sturgeon (*A. baerii* ♀ × *A. schrenckii* ♂) (*n* = 6). Notes: *β-actin* was the internal reference gene. *claudin1*, *claudin2*, *occludin*, *zo1*, *zo2*, and *zo3* represent Claudin-1, Claudin-2, Occludin, Zonula Occludens-1, Zonula Occludens-2, and Zonula Occludens-3, respectively. Different superscript letters in the figure indicate significant differences between groups. The orthogonal polynomial method is used to evaluate the effect of the methionine supplemental level on the parameters of the SPC replacement of FM group, which are divided into linear type and quadratic type. The same applies hereafter.

As shown in Figure 2, compared with the FM group, the mRNA expression levels of claudin1, claudin2, occludin, zo1, zo2, and zo3 in the intestine of hybrid sturgeon significantly decreased in the M0 group (*p* < 0.05). These levels significantly increased in the M5 group compared to the M0 group (*p* < 0.05).

### 3.8. Intestinal Immune-Related Gene Expression

**Figure 3:**
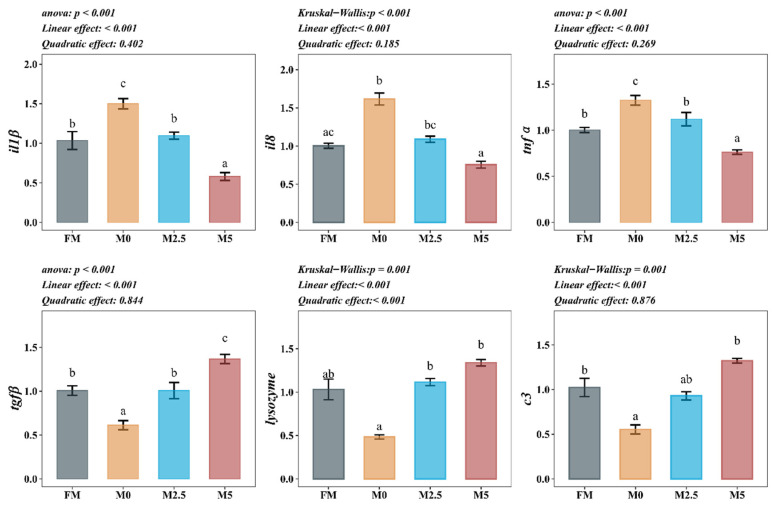
Effects of replacing fish meal with soybean protein concentrate and supplementing methionine on intestinal immune barrier-related gene expression of hybrid sturgeon (*A. baerii* ♀ × *A. schrenckii* ♂) (*n* = 6). Notes: *β-actin* was the internal reference gene. *il1β*, *il8*, *tnfα*, *tgfβ*, *lysozyme*, and *c3* represent Interleukin-1 beta, Interleukin-8, Tumor Necrosis Factor-alpha, Transforming Growth Factor-beta, Lysozyme, and Complement Component 3, respectively. Different superscript letters in the figure indicate significant differences between groups. The orthogonal polynomial method is used to evaluate the effect of the methionine supplemental level on the parameters of the SPC replacement of FM group, which are divided into linear type and quadratic type.

As shown in Figure 3, compared with the FM group, the expression of il8 was significantly upregulated while tgfβ and c3 were significantly downregulated in the M0 group (*p* < 0.05). Compared with the M0 group, the expression of il1β, il8, and tnfα was significantly downregulated while the expression of tgfβ, lysozyme, and c3 was significantly upregulated in the M5 group (*p* < 0.05).

### 3.9. Serum Biochemical Indices

As shown in Table 11, compared with the FM group, the serum levels of TC, HDL-C, and LDL-C significantly increased in the M0 and M2.5 groups, and TG levels as well as GOT and GPT activities significantly increased in the M0 group (*p* < 0.05). Compared with the M0 group, the serum levels of TG, TC, and LDL-C, as well as GOT and GPT activities, significantly decreased while HDL-C levels significantly increased in the M5 group (*p* < 0.05).

### 3.10. Hepatic Antioxidant Indices

As shown in Table 12, compared with the FM group, hepatic MDA levels and CAT and SOD activities significantly increased in the M0, M2.5, and M5 groups (*p* < 0.05). Compared with the M0 group, hepatic MDA levels and CAT and SOD activities significantly decreased in the M2.5 and M5 groups (*p* < 0.05).

### 3.11. Muscle Structure

**Figure 4:**
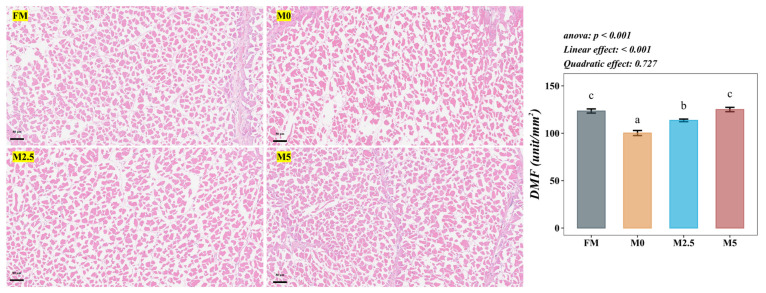
Effects of replacing fish meal with soybean protein concentrate and supplementing methionine on muscle structure of hybrid sturgeon (*A. baerii* ♀ × *A. schrenckii* ♂) (*n* = 6). Notes: Crosscut—H&E staining (×100). DMF represents density of muscle fiber. Different letters indicate that the difference is statistically significant at a significance level of *p* < 0.05. Different superscript letters in the figure indicate significant differences between groups. The orthogonal polynomial method is used to evaluate the effect of the methionine supplemental level on the parameters of the SPC replacement of FM group, which are divided into linear type and quadratic type.

As shown in Figure 4, compared with the FM group, the muscle fiber density of hybrid sturgeon significantly decreased in the M0 group (*p* < 0.05). Compared with the M0 group, muscle fiber density significantly increased in the M5 group (*p* < 0.05).

### 3.12. Muscle Texture Parameters

As shown in Table 13, compared with the FM group, muscle hardness and chewiness significantly decreased in the M0 group (*p* < 0.05). Compared with the M0 group, muscle hardness, adhesiveness, and chewiness significantly increased in the M5 group (*p* < 0.05).

## 4. Discussion

In recent years, soybean protein concentrate (SPC) has emerged as a cost-effective and widely available alternative to fish meal (FM) for high-value aquaculture species, particularly carnivorous fish. Its advantages include a lower content of anti-nutritional factors compared to soybean meal. However, the deficiency in essential amino acids (EAAs), particularly methionine and lysine, remains a key limitation to the extensive use of SPC. Previous studies conducted in our laboratory on *Monopterus albus* (Asian swamp eel) demonstrated that replacing FM with SPC significantly impaired growth performance, while methionine supplementation effectively mitigated this decline [5,6]. Consistent with these findings, the present study revealed that replacing a high proportion of FM with SPC significantly reduced weight gain and protein efficiency while increasing the feed conversion ratio (FCR) in hybrid sturgeon. Supplementation with methionine progressively restored growth performance to levels comparable to the FM group. Three primary mechanisms may explain the observed improvements in growth performance with methionine supplementation. First, methionine is an essential amino acid critical for growth and protein synthesis in fish. Methionine deficiency can limit protein synthesis efficiency, and supplementation can rectify amino acid imbalances in SPC-based diets, thereby enhancing growth [33,34]. Second, methionine, through its conversion to S-adenosylmethionine (SAM), plays a pivotal role in DNA and RNA methylation, which regulates gene expression and cellular proliferation. Methionine supplementation supports these biochemical processes, ensuring normal cell growth and differentiation [35,36]. Third, methionine may promote lipid and energy metabolism, enabling aquaculture species to better utilize dietary energy, which contributes to improved growth [37,38].

Digestive enzyme activity, including amylase, lipase, and trypsin, is a key factor influencing nutrient absorption efficiency in fish. Higher enzyme activity enhances the digestion of carbohydrates, lipids, and proteins, ensuring adequate energy and nutrient availability for growth [39,40,41]. In this study, amylase activity was significantly elevated in the M0 group, while lipase and trypsin activities were significantly reduced compared to the FM group. This may be attributed to differences in the nutritional composition of FM and SPC. SPC typically contains higher carbohydrate levels than FM. To adapt to higher dietary carbohydrate intake, the intestines may increase amylase secretion to improve starch digestion [42]. However, the imbalance of amino acids and fatty acids in SPC may compromise the utilization of dietary proteins and lipids, resulting in reduced lipase and trypsin activities [43]. Similar results have been reported in species such as Amur sturgeon (*Acipenser schrenckii*), yellow catfish (*Pelteobagrus fulvidraco*), Chinese sucker (*Myxocyprinus asiaticus*), and turbot (*Scophthalmus maximus* L.), where FM replacement with SPC decreased lipase and trypsin activities [26,44,45,46]. Methionine supplementation significantly increased lipase and trypsin activities in hybrid sturgeon, aligning with the improved growth performance and intestinal structure observed in this study. Methionine, as an essential amino acid for protein synthesis, likely stimulates the secretion of trypsin, optimizing protein digestion to meet higher amino acid demands. Similar findings have been reported in gilthead seabream (*Sparus aurata* L.), yellow catfish, and grass carp, where methionine supplementation alleviated amino acid imbalances and restored digestive enzyme activity in SPC-based diets [47,48,49].

Tight junction proteins, including *claudin1*, *claudin2*, *occludin*, *zo1*, *zo2*, and *zo3*, are essential for maintaining intestinal epithelial integrity and barrier function [50]. Pro-inflammatory and anti-inflammatory cytokines, such as *il1β*, *il8*, *tnfα*, *tgfβ*, as well as complement component gene *c3* and antimicrobial gene *lysozyme*, collectively regulate intestinal immune responses [51,52]. Although SPC has lower levels of anti-nutritional factors than soybean meal, residual anti-nutritional compounds and amino acid imbalances in SPC may still damage intestinal health [53,54]. In this study, hybrid sturgeon fed SPC without methionine supplementation (M0 group) exhibited significant downregulation of tight junction genes (*claudin1*, *claudin2*, *occludin*, *zo1*, *zo2*, and *zo3*), upregulation of the pro-inflammatory gene *il8*, and reduced expression of *tgfβ* and *c3*. Additionally, goblet cell numbers were reduced, indicating impaired intestinal barrier function. In contrast, methionine supplementation at 0.5% significantly upregulated tight junction gene expression, reduced pro-inflammatory cytokines (*il1β*, *il8*, and *tnfα*), and increased anti-inflammatory (*tgfβ*, *lysozyme*, and *c3*) gene expression. Goblet cell numbers also increased, suggesting improved mucosal barrier integrity. Methionine, as a sulfur-containing amino acid, is essential for cellular growth and repair, enhancing the stability of the intestinal barrier [55]. Additionally, methionine contributes to methylation and glutathione synthesis, exerting antioxidant and immunomodulatory effects that reduce oxidative stress and inflammation [56]. The above-mentioned studies may provide an explanation for the results observed in this experiment.

The liver plays a critical role in lipid synthesis, breakdown, and storage. In this study, replacing FM with SPC led to significant increases in the HSI and VSI in groups without methionine supplementation (M0), while methionine supplementation (M2.5 and M5) reduced both indices. An elevated HSI is typically associated with excessive lipid accumulation in the liver, potentially caused by high levels of dietary carbohydrates or fats, leading to increased lipid storage [57]. Conversely, reductions in the HSI often indicate decreased hepatic lipid reserves, likely due to improved energy utilization and mobilization of stored lipids for energy supply. These findings suggest that methionine supplementation could potentially enhance lipid metabolism [58]. Methionine supplementation was also associated with significant increases in whole-body crude protein content and reductions in whole-body crude lipid content compared to the M0 group. Similar results have been observed in cobia, pufferfish (*Takifugu rubripes*), and yellowtail (*Seriola dorsalis*) [59,60,61]. This could be attributed to methionine’s ability to optimize energy metabolism, promoting protein synthesis and accumulation while facilitating lipid breakdown and utilization. Such effects help reduce hepatic or systemic lipid deposition, ultimately resulting in a lower crude lipid content [13].

Serum biochemical indices are crucial indicators of lipid metabolism [62]. In this study, SPC diets led to significant increases in serum TC, HDL-C, and LDL-C levels, as well as TG and transaminase (GOT and GPT) activities, particularly in the M0 group. Methionine deficiency likely increased metabolic pressure on the liver, leading to abnormal lipid metabolism and greater metabolic burden, potentially impairing liver function [63]. Conversely, methionine supplementation in the M5 group significantly reduced serum TG, TC, and LDL-C levels and GOT/GPT activities while increasing HDL-C levels. These changes indicate that methionine supplementation restored normal lipid metabolism, reduced hepatic metabolic stress, and improved liver function, which aligns with both the morphological indices and body composition results observed in this study. Liver MDA, CAT, and SOD levels are key markers of oxidative stress and antioxidant capacity [64]. Methionine is metabolized into cysteine, a precursor for glutathione synthesis [65]. Additionally, methionine contributes to the synthesis of SAM, which participates in methylation reactions that regulate gene expression, repair DNA damage, and enhance antioxidant enzyme activity [66]. Studies on European seabass (*Dicentrarchus labrax*), rohu (*Labeo rohita*), and spotted seabass (*Lateolabrax maculatus*) have shown that methionine supplementation reduces lipid peroxidation, protecting cell membrane stability and integrity. It also reduces oxidative stress-induced inflammatory responses, thereby mitigating tissue damage caused by oxidative stress [67,68,69]. Consistent with these findings, this study demonstrated that methionine supplementation significantly reduced hepatic MDA levels while enhancing CAT and SOD activities in hybrid sturgeon. These results suggest that methionine may protect cells from oxidative stress by promoting the activity of antioxidant enzymes, thus reducing hepatic oxidative damage.

In aquatic animals, muscle texture parameters such as hardness and cohesiveness are structural indicators reflecting the firmness and structural integrity of the muscle tissue. Elasticity, on the other hand, measures the ability of the muscle to regain its shape after chewing, which is an important indicator of freshness. Adhesiveness and gumminess represent the adherence properties of the muscle, describing whether the muscle tissue easily sticks to the oral cavity during chewing and its smoothness during swallowing. Chewiness, a comprehensive indicator, reflects the effort required to chew the muscle to a swallowable state and combines hardness, cohesiveness, and elasticity [70,71]. Muscle fiber density is closely linked to growth rates and nutritional status. High fiber density generally indicates an optimal growth environment and adequate nutrient supply, whereas low-density or excessively coarse fibers may suggest suboptimal nutrition or excessively rapid growth [72,73]. In this study, compared to the FM group, hybrid sturgeon in the M0 group (SPC-based diet without methionine supplementation) showed significantly reduced muscle hardness, chewiness, and fiber density. Conversely, methionine supplementation in the M5 group significantly increased muscle hardness, gumminess, chewiness, and fiber density compared to the M0 group. The observed decline in muscle hardness, chewiness, and fiber density in the M0 group can be attributed to methionine deficiency. As an essential amino acid required for protein synthesis, methionine is critical for muscle fiber formation and maintenance. Its deficiency can result in loose muscle structure and reduced hardness [14]. Supplementing methionine in the M5 group enhanced protein synthesis efficiency, increased muscle fiber density, and tightened muscle tissue structure, leading to significant improvements in muscle hardness, gumminess, and chewiness. Additionally, adequate methionine improves water retention in muscle tissues, contributing to enhanced elasticity [20]. Similar findings have been reported in studies on gilthead seabream (*Sparus aurata*) and grass carp, where methionine supplementation improved muscle quality by enhancing muscle fiber structure and density, leading to increased hardness and chewiness [74,75].

## 5. Conclusions

Replacing FM with SPC impaired growth performance, intestinal health, liver lipid metabolism, and muscle quality in hybrid sturgeon. Methionine supplementation mitigated these negative effects, restoring growth performance, improving intestinal barrier and immune function, normalizing liver metabolism, and enhancing muscle texture and density. Optimal methionine supplementation levels effectively promote the health and growth of SPC-fed hybrid sturgeon.

## Figures and Tables

**Table 1:** Nutrient levels of fish meal and soy protein concentrate (%).

Items	Fish Meal	Soy Protein Concentrate
Moisture	8.06	8.89
Crude protein	55.56	66.72
Crude fat	9.51	0.14
Ash	16.38	5.73
Asp	7.41	7.81
Glu	7.56	12.48
Ser	3.54	3.49
Gly	4.33	2.91
Ala	4.04	2.95
Tyr	2.42	2.55
Pro	4.72	4.48
Cys	1.40	1.36
* His	1.63	1.67
* Thr	2.58	2.75
* Arg	4.63	4.85
* Val	2.97	3
* Met	1.68	0.63
* Phe	3.48	3.65
* Ile	3.55	3.25
* Leu	5.38	5.4
* Lys	4.57	2.27
EAA	26.25	23.05
TAA	65.90	65.50

Notes: * is essential amino acid. EAA and TAA represent essential amino acids and total amino acids, respectively. The same applies hereafter.

**Table 2:** Composition of the diets and nutrition level (dry matter) (%).

Items	Groups			
	FM	M0	M2.5	M5
Fish meal	39.50	22.50	22.50	22.50
Soy protein concentrate	0.00	14.00	14.00	14.00
Poultry meal	14.00	14.00	14.00	14.00
Fermented soybean meal	6.00	6.00	6.00	6.00
Soybean meal	9.00	9.00	9.00	9.00
Rapeseed meal	8.00	8.00	8.00	8.00
Wheat flour	12.70	12.70	12.70	12.70
Fish oil	0.00	2.00	2.00	2.00
Soy oil	8.30	8.00	8.00	8.00
Ca(H_2_PO_4_)_2_	1.50	1.50	1.50	1.50
DL-Methionine	0.00	0.00	0.25	0.50
Lysine	0.00	0.25	0.25	0.25
Glutamate	0.50	0.00	0.00	0.00
Glycine	0.00	0.25	0.25	0.25
Alanine	0.00	0.25	0.25	0.25
Premix	0.30	0.30	0.30	0.30
Bentonite	0.20	1.25	1.00	0.75
Total	100.00	100.00	100.00	100.00
Nutrient levels				
Crude protein	46.58	46.02	46.10	45.81
Crude fat	12.68	12.73	12.87	12.72
Ash	9.32	7.17	7.11	7.24

Note—each kilogram of premix provides feed with the following: VA 300,000 IU, VD_3_ 150,000 IU, VE 4000 mg, VK_3_ 450 mg, VB_1_ 800 mg, VB_2_ 850 mg, VB_6_ 600 mg, VB_12_ 1.5 mg, Inositol 4000 mg, VC 11,000 mg, nicotinamide 3000 mg, D-calcium pantothenate 1700 mg, folate 130 mg, D-biotin 15 mg, copper 650 mg, iron 4500 mg, manganese 850 mg, zinc 5300 mg, Iodine 120 mg, selenium 35 mg, cobalt 100 mg.

**Table 3:** Amino acid composition of the diets (%).

Items	Groups			
	FM	M0	M2.5	M5
Asp	4.11	4.09	4.07	4.13
Glu	6.74	6.71	6.69	6.76
Ser	1.92	1.89	1.89	1.93
Gly	2.78	2.80	2.81	2.84
Ala	2.58	2.56	2.59	2.54
Tyr	1.36	1.38	1.42	1.38
Pro	2.37	2.33	2.34	2.37
* His	1.12	1.17	1.18	1.19
* Thr	1.71	1.67	1.67	1.69
* Arg	2.80	2.80	2.83	2.86
* Val	2.10	2.04	2.05	2.03
* Met	0.84	0.61	0.88	1.21
* Phe	1.99	2.02	2.04	1.96
* Ile	1.73	1.74	1.74	1.70
* Leu	3.20	3.21	3.22	3.17
* Lys	2.99	3.01	3.02	3.00
EAA	18.49	18.26	18.62	18.81
TAA	40.34	40.02	40.43	40.76

Notes: * is essential amino acid. Cystine is not detected.

**Table 4:** Primers of intestinal physical barrier- and immune barrier-related genes of hybrid sturgeon (*A. baerii* ♀ × *A. schrenckii* ♂).

Gene	Forward Primer (5′-3′)	Reverse Primer (5′-3′)	Accession No.
*β-actin*	CGGTTTCGCTGGAGATGATG	TCAGTGAGCAGGACGGGGTG	JX027376.1
*claudin1*	AGACTAGAGCCATGCCCTCA	GGTGCACGACTGTGAATACG	XM_034041590.3
*claudin2*	TGTCTGCCTTGCTCTCCTTT	CTTTGCTCTGGGGATGGTTA	XM_034000284.3
*claudin3*	TAGGCAGCATTGTGGCTTGCAC	AGCCAGCATGGAGTCGTAGACC	XM_034915357.2
*occludin*	TCCGATCAGTGGAGACCAGCAG	TCCAGCCGAGACAGGTCCTTG	XM_058986519.1
*zo1*	AAGCACGGCCTCGTTATGAACC	TGGAGGTGGTGGCAGAGCAG	XM_058998822.1
*zo2*	TCTCACTCCAGCAGCCAGAGC	CACTGCCGGATCATGGACACTG	XM_058993402.1
*zo3*	GGAGGAGGATCTCGACGCTCTG	CGCCTCTCCGTCCGTGTCC	XM_059013834.1
*il1β*	ACATTGCCAACCTCATCATCG	TTGAGCAGGTCCTTGTCCTTG	AJ223954
*il8*	AGAATGTCAGCCAGCCTTGT	TCTCAGACTCATCCCCTCAGT	AJ279069
*tnfα*	TGGAGGGGTATGCGATGACACCTG	TGAGGCCTTTCTCTCAGCGACAGC	AJ249755.1
*tgfβ*	GCAGCTGTTCTTCAACATGT	GTGCCCTTGTACAGCTCTAT	GQ205390.1
*lysozyme*	TGGAAGTGGTGTTTTTGTGT	TCAAATCCATCAAGCCCTTC	MF135537.1
*c3*	ATGAGCTCCTGCAGAGGTGT	AGTGGTTGTTGGAGGTCTGG	XM_059028619.1

Notes: *β-actin*—Beta-actin; *claudin1*—Claudin-1; *claudin2*—Claudin-2; *claudin3*—Claudin-3; *occludin*—Occludin; *zo1*—Zonula Occludens-1; *zo2*—Zonula Occludens-2; *zo3*—Zonula Occludens-3; *il1β*—Interleukin-1 beta; *il8*—Interleukin-8; *tnfα*—Tumor Necrosis Factor-alpha; *tgfβ*—Transforming Growth Factor-beta; *lysozyme*—Lysozyme; *c3*—Complement Component 3.

**Table 5:** Effects of replacing fish meal with soybean protein concentrate and supplementing methionine on growth performance of hybrid sturgeon (*A. baerii* ♀ × *A. schrenckii* ♂) (*n* = 3).

Items	Groups				*p*		
	FM	M0	M2.5	M5	*p*	L	Q
W_0_	8.00 ± 0.00	8.00 ± 0.00	8.00 ± 0.00	8.00 ± 0.00	/	/	/
W_t_	30.57 ± 0.2 ^a^	26.02 ± 0.33 ^c^	28.75 ± 0.4 ^b^	29.39 ± 0.26 ^ab^	<0.01	<0.001	0.042
WGR	282.07 ± 2.48 ^a^	225.22 ± 4.08 ^c^	259.42 ± 5.03 ^b^	267.36 ± 3.19 ^ab^	<0.01	<0.001	0.042
FCR	1.23 ± 0.02 ^b^	1.52 ± 0.05 ^a^	1.32 ± 0.03 ^b^	1.26 ± 0.01 ^b^	<0.01	0.420	0.635
SR	97.78 ± 1.11	98.89 ± 1.11	98.89 ± 1.11	100.0 ± 0.0	0.432	0.003	0.183
PER	2.39 ± 0.03 ^a^	1.94 ± 0.07 ^b^	2.23 ± 0.06 ^a^	2.33 ± 0.03 ^a^	<0.01	0.002	0.212

Notes: Weight gain rate (WGR, %) = (W_t_ − W_0_)/W_0_ × 100%; feed conversion ratio (FCR) = F/(W_t_ − W_0_); survival rate (SR, %) = N_t_/N_0_ × 100%; protein efficiency ratio (PER, %) = (Wt − W_0_)/*p*; where N_0_ is the initial number; N_t_ is the end number; W_0_ is the initial body weight (g); W_t_ is the end body weight (g); F is the average feed intake feed amount (g); *p* is the total protein intake (g). Values in each item with the different superscript of superscripts are significant different. The orthogonal polynomial method is used to evaluate the effect of the methionine supplemental level on the parameters of the SPC replacement of FM group, which are divided into linear type and quadratic type. The same applies hereafter.

**Table 6:** Effects of replacing fish meal with soybean protein concentrate and supplementing methionine on morphological indices of hybrid sturgeon (*A. baerii* ♀ × *A. schrenckii* ♂) (*n* = 6).

Items	Groups				*p*		
	FM	M0	M2.5	M5	*p*	L	Q
HSI	2.92 ± 0.11 ^b^	4.73 ± 0.45 ^a^	3.59 ± 0.25 ^a^	3.2 ± 0.33 ^ab^	0.021	0.008	0.408
VSI	10.26 ± 0.31 ^b^	12.64 ± 0.4 ^a^	10.84 ± 0.14 ^b^	10.58 ± 0.23 ^b^	<0.01	<0.001	0.039
CF	6.02 ± 0.13	6.17 ± 0.3	5.94 ± 0.17	5.88 ± 0.12	0.873	0.327	0.742

Notes: Hepatosomatic index (HSI, %) = W_h_/W_t_ × 100%; viscerosomatic index (VSI, %) = W_v_/W_t_ × 100%; condition factor (CF, g/cm^3^) = W_t_/L^3^ × 100; where W_t_ is the end body weight (g); W_h_ is the liver weight (g); W_v_ is the visceral weight (g); L is the body length (cm). Values in each item with the different superscript of superscripts are significant different. The orthogonal polynomial method is used to evaluate the effect of the methionine supplemental level on the parameters of the SPC replacement of FM group, which are divided into linear type and quadratic type.

**Table 7:** Effects of replacing fish meal with soybean protein concentrate and supplementing methionine on nutritional composition of whole fish of hybrid sturgeon (*A. baerii* ♀ × *A. schrenckii* ♂) (%) (*n* = 3).

Items	Groups				*p*		
	FM	M0	M2.5	M5	*p*	L	Q
Moisture	80.43 ± 0.61	80.42 ± 0.01	80.38 ± 0.01	80.33 ± 0.01	0.183	<0.001	1.000
Crude protein	12.5 ± 0.23 ^a^	11.54 ± 0.13 ^b^	11.76 ± 0.23 ^ab^	12.15 ± 0.16 ^a^	0.03	0.052	0.707
Crude fat	4.11 ± 0.46	4.73 ± 0.08	4.58 ± 0.34	4.12 ± 0.35	0.369	0.179	0.674
Ash	2.68 ± 0.04	2.47 ± 0.02	2.41 ± 0.13	2.54 ± 0.07	0.15	0.567	0.402

Note: Values in each item with the different superscript of superscripts are significant different. The orthogonal polynomial method is used to evaluate the effect of the methionine supplemental level on the parameters of the SPC replacement of FM group, which are divided into linear type and quadratic type.

**Table 8:** Effects of replacing fish meal with soybean protein concentrate and supplementing methionine on amino acid composition of whole fish of hybrid sturgeon (*A. baerii* ♀ × *A. schrenckii* ♂) (%) (*n* = 3).

Items	Groups				*p*		
	FM	M0	M2.5	M5	*p*	L	Q
Asp	1.07 ± 0.01	1.03 ± 0.02	1.04 ± 0.02	1.06 ± 0.04	0.65	0.438	0.850
Glu	1.59 ± 0.01	1.53 ± 0.05	1.56 ± 0.03	1.61 ± 0.06	0.6	0.303	0.970
Ser	0.55 ± 0.01	0.53 ± 0.01	0.53 ± 0.01	0.54 ± 0.01	0.29	0.276	0.970
Gly	1.18 ± 0.03	1.17 ± 0.03	1.18 ± 0.01	1.17 ± 0.01	0.95	0.847	0.620
Ala	0.79 ± 0.02	0.78 ± 0.02	0.77 ± 0.01	0.79 ± 0.02	0.87	0.640	0.692
Tyr	0.38 ± 0.01	0.35 ± 0.01	0.36 ± 0.01	0.41 ± 0.03	0.319	0.084	0.483
* His	0.24 ± 0.01	0.24 ± 0.0	0.24 ± 0.0	0.24 ± 0.01	0.7	0.949	0.312
* Thr	0.5 ± 0.0	0.49 ± 0.01	0.5 ± 0.01	0.51 ± 0.02	0.63	0.301	0.886
* Arg	0.77 ± 0.01	0.74 ± 0.03	0.77 ± 0.01	0.75 ± 0.02	0.64	0.876	0.338
* Val	0.53 ± 0.0	0.52 ± 0.01	0.53 ± 0.02	0.56 ± 0.02	0.37	0.162	0.789
* Met	0.29 ± 0.0	0.28 ± 0.01	0.29 ± 0.01	0.3 ± 0.01	0.4	0.148	0.900
* Phe	0.45 ± 0.01	0.42 ± 0.01	0.43 ± 0.01	0.43 ± 0.01	0.32	0.431	0.710
* Ile	0.49 ± 0.01	0.45 ± 0.01	0.46 ± 0.01	0.48 ± 0.02	0.26	0.222	0.966
* Leu	0.81 ± 0.01	0.78 ± 0.01	0.79 ± 0.02	0.83 ± 0.02	0.28	0.130	0.614
* Lys	0.86 ± 0.02	0.82 ± 0.01	0.85 ± 0.02	0.85 ± 0.04	0.75	0.435	0.820
NEAA	4.92 ± 0.01	4.75 ± 0.1	4.87 ± 0.1	4.95 ± 0.15	0.58	0.292	0.909
TAA	17.27 ± 0.38	17.33 ± 0.22	17.31 ± 0.42	17.18 ± 0.56	0.55	0.285	0.983

Notes: * is essential amino acid. Cystine and proline are not detected. The orthogonal polynomial method is used to evaluate the effect of the methionine supplemental level on the parameters of the SPC replacement of FM group, which are divided into linear type and quadratic type.

**Table 9:** Effects of replacing fish meal with soybean protein concentrate and supplementing methionine on intestinal digestive enzymes of hybrid sturgeon (*A. baerii* ♀ × *A. schrenckii* ♂) (*n* = 6).

Items	Groups				*p*		
	FM	M0	M2.5	M5	*p*	L	Q
AMS (U/g prot)	0.23 ± 0.01 ^b^	0.31 ± 0.01 ^a^	0.31 ± 0.01 ^a^	0.32 ± 0.01 ^a^	<0.01	0.442	0.173
LPS (U/g prot)	10.36 ± 0.15 ^a^	7.15 ± 0.14 ^c^	9.32 ± 0.12 ^b^	10.65 ± 0.11 ^a^	<0.01	<0.001	0.015
TPS (U/µg prot)	7.09 ± 0.11 ^a^	6.05 ± 0.13 ^b^	6.36 ± 0.17 ^b^	7.34 ± 0.14 ^a^	<0.01	<0.001	0.085

Note: AMS, LPS, and TPS represent amylase, lipopolysaccharide, and trypsin, respectively. Values in each item with the different superscript of superscripts are significant different. The orthogonal polynomial method is used to evaluate the effect of the methionine supplemental level on the parameters of the SPC replacement of FM group, which are divided into linear type and quadratic type.

**Table 10:** Effects of replacing fish meal with soybean protein concentrate and supplementing methionine on intestinal tissue structure of hybrid sturgeon (*A. baerii* ♀ × *A. schrenckii* ♂) (*n* = 6).

Items	Groups				*p*		
	FM	M0	M2.5	M5	*p*	L	Q
VH (μm)	562.15 ± 3.66 ^b^	504.66 ± 6.76 ^c^	564.28 ± 5.14 ^b^	619.02 ± 3.88 ^a^	<0.01	<0.001	0.717
MT (μm)	73.15 ± 0.75	71.89 ± 0.96	70.91 ± 1.07	73.44 ± 0.52	0.16	0.234	0.125
GC (unit/root)	42.17 ± 0.48 ^b^	36.33 ± 0.21 ^c^	40.83 ± 0.75 ^b^	45.83 ± 0.65 ^a^	<0.01	<0.001	0.733

Note: VH, MT, and GC represent intestinal villus height, muscle thickness, and goblet cells, respectively. Values in each item with the different superscript of superscripts are significant different. The orthogonal polynomial method is used to evaluate the effect of the methionine supplemental level on the parameters of the SPC replacement of FM group, which are divided into linear type and quadratic type.

**Table 11:** Effects of replacing fish meal with soybean protein concentrate and supplementing methionine on serum biochemical indices of hybrid sturgeon (*A. baerii* ♀ × *A. schrenckii* ♂) (*n* = 6).

Items	Groups				*p*		
	FM	M0	M2.5	M5	*p*	L	Q
TG (mmol/L)	7.86 ± 0.04 ^b^	8.6 ± 0.12 ^a^	8.09 ± 0.08 ^ab^	7.97 ± 0.09 ^b^	<0.01	<0.001	0.131
TC (mmol/L)	3.25 ± 0.05 ^b^	3.64 ± 0.06 ^a^	3.6 ± 0.08 ^a^	3.26 ± 0.08 ^b^	<0.01	0.002	0.113
HDL-C (mmol/L)	0.58 ± 0.01 ^c^	0.6 ± 0.01 ^b^	0.61 ± 0.01 ^b^	0.66 ± 0.01 ^a^	<0.01	<0.001	0.019
LDL-C (mmol/L)	1.51 ± 0.04 ^b^	1.74 ± 0.04 ^a^	1.71 ± 0.06 ^a^	1.34 ± 0.03 ^c^	<0.01	<0.001	0.004
GOT (U/L)	163.47 ± 2.72 ^b^	202.63 ± 3.38 ^a^	170.72 ± 1.26 ^b^	159.21 ± 3.73 ^b^	<0.01	<0.001	0.014
GPT (U/L)	50.34 ± 0.92 ^b^	61.52 ± 1.93 ^a^	53.8 ± 1.03 ^b^	50.82 ± 0.85 ^b^	<0.01	<0.001	0.174

Note: TG, TC, HDL-C, LDL-C, GOT, and GPT represent triacylglycerol, total cholesterol, high-density lipoprotein cholesterol, low-density lipoprotein cholesterol, glutamic oxaloacetic transaminase, and glutamic pyruvic transaminase, respectively. Values in each item with the different superscript of superscripts are significant different. The orthogonal polynomial method is used to evaluate the effect of the methionine supplemental level on the parameters of the SPC replacement of FM group, which are divided into linear type and quadratic type.

**Table 12:** Effects of replacing fish meal with soybean protein concentrate and supplementing methionine on liver antioxidant indices of hybrid sturgeon (*A. baerii* ♀ × *A. schrenckii* ♂) (*n* = 6).

Items	Groups				*p*		
	FM	M0	M2.5	M5	*p*	L	Q
MDA (nmol/mg prot)	4.62 ± 0.03 ^d^	6.64 ± 0.04 ^a^	5.62 ± 0.03 ^b^	4.77 ± 0.03 ^c^	<0.01	<0.001	0.055
CAT (U/mg prot)	8.22 ± 0.13 ^c^	10.7 ± 0.02 ^a^	8.9 ± 0.09 ^b^	8.68 ± 0.05 ^b^	<0.01	<0.001	<0.001
SOD (U/mg prot)	24.03 ± 0.63 ^c^	34.72 ± 0.48 ^a^	27.61 ± 0.61 ^b^	27.53 ± 0.76 ^b^	<0.01	<0.001	<0.001

Note: MDA, CAT, and SOD represent malondialdehyde, catalase, and superoxide dismutase, respectively. Values in each item with the different superscript of superscripts are significant different. The orthogonal polynomial method is used to evaluate the effect of the methionine supplemental level on the parameters of the SPC replacement of FM group, which are divided into linear type and quadratic type.

**Table 13:** Effects of replacing fish meal with soybean protein concentrate and supplementing methionine on textural parameters of hybrid sturgeon (*A. baerii* ♀ × *A. schrenckii* ♂) (*n* = 6).

Items	Groups				*p*		
	FM	M0	M2.5	M5	*p*	L	Q
Hardness (N)	6.31 ± 0.2 ^a^	5.1 ± 0.36 ^b^	6.27 ± 0.14 ^ab^	7.15 ± 0.43 ^a^	<0.01	<0.001	0.734
Adhesiveness (×10, N.mm)	0.45 ± 0.04	0.43 ± 0.02	0.44 ± 0.03	0.43 ± 0.04	0.98	0.961	0.899
Cohesiveness (%)	0.42 ± 0.03	0.41 ± 0.02	0.41 ± 0.02	0.41 ± 0.01	0.97	0.993	0.677
Springiness (mm)	1.1 ± 0.03	1.05 ± 0.01	1.08 ± 0.09	1.15 ± 0.05	0.483	0.204	0.794
Gumminess (N)	2.51 ± 0.17 ^ab^	1.92 ± 0.25 ^b^	3.25 ± 0.21 ^a^	3.63 ± 0.43 ^a^	<0.01	0.002	0.232
Chewiness (mJ)	3.43 ± 0.18 ^a^	1.89 ± 0.25 ^b^	3.24 ± 0.19 ^a^	3.64 ± 0.32 ^a^	<0.01	<0.001	0.154

Note: Values in each item with the different superscript of superscripts are significant different. The orthogonal polynomial method is used to evaluate the effect of the methionine supplemental level on the parameters of the SPC replacement of FM group, which are divided into linear type and quadratic type.

## Data Availability

The datasets used and/or analyzed in the current study are available from the corresponding author on reasonable request.
